# Life-course origins of social inequalities in adult immune cell markers of inflammation in a developing southern Chinese population: the Guangzhou Biobank Cohort Study

**DOI:** 10.1186/1471-2458-12-269

**Published:** 2012-04-03

**Authors:** Douglas A West, Gabriel M Leung, Chao Q Jiang, Timothy M Elwell-Sutton, Wei S Zhang, Tai H Lam, Kar K Cheng, C Mary Schooling

**Affiliations:** 1 School of Public Health, Li Ka Shing Faculty of Medicine, The University of Hong Kong, 21 Sassoon Road, Pokfulam, Hong Kong, SAR, China; 2 Guangzhou Occupational Diseases Prevention and Treatment Centre, Guangzhou Number 12 Hospital, Guangzhou, China; 3 Department of Public Health and Epidemiology, University of Birmingham, Birmingham, UK

## Abstract

**Background:**

Socioeconomic position (SEP) throughout life is associated with cardiovascular disease, though the mechanisms linking these two are unclear. It is also unclear whether there are critical periods in the life course when exposure to better socioeconomic conditions confers advantages or whether SEP exposures accumulate across the whole life course. Inflammation may be a mechanism linking socioeconomic position (SEP) with cardiovascular disease. In a large sample of older residents of Guangzhou, in southern China, we examined the association of life course SEP with inflammation.

**Methods:**

In baseline data on 9,981 adults (≥ 50 years old) from the Guangzhou Biobank Cohort Study (2006-08), we used multivariable linear regression and model fit to assess the associations of life course SEP at four stages (childhood, early adult, late adult and current) with white blood, granulocyte and lymphocyte cell counts.

**Results:**

A model including SEP at all four life stages best explained the association of life course SEP with white blood and granulocyte cell count for men and women, with early adult SEP (education) making the largest contribution. A critical period model best explained the association of life course SEP with lymphocyte count, with sex-specific associations. Early adult SEP was negatively associated with lymphocytes for women.

**Conclusions:**

Low SEP throughout life may negatively impact late adult immune-inflammatory status. However, some aspects of immune-inflammatory status may be sensitive to earlier exposures, with sex-specific associations. The findings were compatible with the hypothesis that in a developing population, upregulation of the gonadotropic axis with economic development may obscure the normally protective effects of social advantage for men.

## Background

Socioeconomic position (SEP) throughout life is usually inversely associated with morbidity and mortality from cardiovascular disease, although the underlying biological pathway is not entirely clear [1,2]. Cardiovascular disease has been associated with higher levels of inflammatory molecules, perhaps as a consequence of exposure to pathogenic organisms [3], although it is unclear whether pathogen burden mediates SEP differences in cardiovascular risk [3,4]. Poor early life conditions are usually associated with higher levels of inflammatory markers [5-11] and poorer adult immune function [12,13]. These associations are less clear, however, amongst men from middle income countries [10]. Furthermore, little is known about the association of SEP across the life course with immune function. The duration or number of exposures across the life course may be most important (the accumulation hypothesis) [14]. Alternatively, the timing of exposure to poor socioeconomic conditions may be crucial as a number of sensitive periods or simply as a single critical period (the critical period hypothesis). It is also possible that either inter- or intra-generational social mobility plays a part.

Developmental trade-offs between growth, maintenance, and reproduction may occur when there are competing demands for energy resources between biological systems [13,15,16], potentially at the expense of immune function in resource-poor environments. Alternatively, intergenerationally and environmentally driven up-regulation of the gonadotropic axis with economic development may obscure some of the normally protective effects of social advantage in the first few generations of men to experience better living conditions [17,18], thus generating epidemiologically stage specific associations between SEP and immune-related functions, such as pro-inflammatory states, among men [18,19].

Rapidly developing mega-cities of China may provide a sentinel for the changes in non-communicable diseases expected with economic development and inform effective interventions to reduce the disease burden. In a large sample of older residents from one of the most developed mega-cities in China, Guangzhou in southern China, we assessed the association of SEP at four life stages with proxies of inflammation (total white blood cell, granulocyte, and lymphocyte counts) and compared models representing the accumulation, sensitive periods and critical period hypotheses. Additionally, we hypothesise that 1) higher life course SEP is protective for adult inflammation, 2) the normal protective effect of social advantage is obscured in men experiencing rapid socioeconomic development.

## Methods

### Sources of data

The Guangzhou Biobank Cohort Study is a collaboration between the Guangzhou No. 12 Hospital (Guangzhou, China) and the universities of Hong Kong (Hong Kong, China) and Birmingham (Birmingham, United Kingdom). The study has been described previously in detail [20]. Participants were drawn from the Guangzhou Health and Happiness Association for the Respectable Elders (GHHARE), a community social and welfare association unofficially aligned with the municipal government, where membership is open to anyone aged 50 years or older for a nominal monthly fee of 4 yuan (US $0.50). Approximately 7 percent of permanent Guangzhou residents aged 50 years or more are members of the GHHARE. Eleven percent of the members were included in this study, who were capable of consenting, were ambulatory, and were not receiving treatments which if discontinued might have resulted in immediate, life-threatening risk, such as chemotherapy, radiotherapy or dialysis. Those with less serious chronic illnesses or with acute illnesses were not specifically excluded from the study though they may have been less likely to attend. Participants were recruited in three phases and this study includes participants recruited in phase 3 only (recruited between 2006 and 2008), because only phase 3 has detailed information on childhood socioeconomic position and inflammatory markers.

Participants underwent a detailed half-day medical interview, as well as a physical examination with fasting blood being sampled. Quantitative haematological analysis was performed using a SYSMEX KX-21 haematology analyser. The Guangzhou Medical Ethics Committee of the Chinese Medical Association approved the study and all participants gave written, informed, consent prior to participation.

### Socioeconomic position across the life course

We used indicators of SEP at four life stages: childhood, early adult, late adult and current SEP. Childhood SEP was measured by an index of notable parental possessions that were appropriate to China in the mid-20th century, based on sociologic accounts of life in southern China at that time [17]. The items selected were a watch, a sewing machine, and a bicycle. These items were categorized, as previously, as none or at least one [21]. As in other similar studies, we used education and longest-held occupation as proxies for early and late adult SEP [22]. Early adult SEP was assessed from the highest level of education (primary or less versus secondary or more). Occupation was categorised as manual (agricultural work, factory work, or sales and service) or non-manual (administrative/managerial, professional/technical, or military/police). Current SEP was assessed from household income per head. Household income was recorded in six categories (<5,000 Yuan, 5000-9,999 Yuan, 10,000-19,999 Yuan, 20,000-29,999 Yuan, 30,000-49,999 Yuan and ≥50,000 Yuan). Household income per head was estimated using the mid-point of each income category and assuming that those in the highest category had an annual income of 75,000 Yuan. The median household income per head was used as the cut-off point between low and high SEP.

### Outcome measures

The primary outcome was total white blood cell count used, as in other studies, as a marker of a pro-inflammatory state [5], and less well functioning immune system. As we do not have a detailed breakdown of different white blood cell types, such as macrophages, we also considered granulocyte and lymphocyte counts as outcomes because these immune cell sub-populations largely relate to innate and adaptive immunity respectively. They have previously been used as markers of inflammation [23,24]. Other measures of inflammation (e.g. C-reactive protein) were not available.

### Statistical analysis

Multivariable linear regression was used to assess the adjusted associations of SEP with the outcomes. Following Mishra et al. [25] we determined the most parsimonious representation of life course SEP by comparing models for three different life course hypotheses (the accumulation, sensitive periods and critical period hypotheses) to a 'fully saturated' model which represents all possible life course SEP trajectories. As in previous work [26], the accumulation hypothesis was represented by a model representing the number of life stages with high socioeconomic position, and the sensitive periods hypothesis by a model in which all four measures of SEP were considered as separate items in one model adjusted for all four measures of SEP. The critical period hypothesis was represented by models in which only one SEP exposure (the critical period) was included [25]. We used the Akaike Information Criterion (AIC) to compare models [27]. A smaller AIC indicates a better model.

We examined whether the outcomes had different associations with SEP by sex or age, from the heterogeneity across subgroups and the significance of an interaction term obtained from a model including all interaction terms with age or sex. All models were adjusted for age (in 5 year age groups) and sex. A second set of models was additionally adjusted for lifestyle factors (smoking, alcohol use, and physical activity categorized as in Table 1) as potential mediators and a third set of models additionally adjusted for body mass index (BMI) as a potential mediator

**Table 1:** Characteristics (mean value or percentage) of 9,981 Chinese adults (≥ 50 years) by socioeconomic position at four life stages in women and men from the Guangzhou Biobank Cohort Study, phase 3, 2006-2008

		Socio-economic position in:														
		**Childhood(Parental possession of a watch, sewing machine, and bicycle)**				Early Adulthood (Education*)			**Late Adulthood (Occupation)**				**Current (Household Income per Head)**			

	**No.**	**0 items**	**1+ items**	**Unknown**	***p *†**	**Low**	**High**	***p *†**	**Manual**	**Non-manual**	**Unknown**	***p *†**	**Low**	**High**	**Unknown**	***p *†**

***Women***																

Number of participants	7445	4084	3123	238		3057	4386		5225	1211	1009		3025	3141	1279	
*Mean (SD)*																
White blood cells (10^9^/L)	7445	6.38(1.5)	6.13(1.5)	6.36(1.4)	<0.001	6.52(1.6)	6.10(1.5)	<0.001	6.33(1.5)	6.04(1.4)	6.25(1.5)	<0.001	6.38(1.6)	6.12(1.5)	6.41(1.6)	<0.001
Granulocytes (10^9^/L)	7445	3.79(1.2)	3.62(1.2)	3.71(1.1)	<0.001	3.89(1.2)	3.60(1.1)	<0.001	3.76(1.2)	3.53(1.1)	3.71(1.2)	<0.001	3.79(1.2)	3.60(1.1)	3.84(1.2)	<0.001
Lymphocytes (10^9^/L)	7445	2.19(0.6)	2.15(0.6)	2.26(0.6)	<0.001	2.22(0.6)	2.14(0.6)	<0.001	2.18(0.6)	2.15(0.6)	2.18(0.6)	0.227	2.20(0.6)	2.16(0.6)	2.16(0.6)	0.008
Leg length (cm)	7430	69.7(3.8)	69.8(3.6)	69.8(4.3)	0.393	69.6(3.8)	69.8(3.7)	0.008	69.7(3.7)	69.9(3.6)	69.6(3.8)	0.202	69.6(3.8)	69.8(3.6)	69.6(3.8)	0.099
Sitting height (cm)	7431	83.7(3.6)	85.1(3.1)	84.1(3.7)	<0.001	83.1(3.6)	85.1(3.1)	<0.001	84.0(3.5)	85.0(3.3)	84.6(3.3)	<0.001	83.9(3.5)	85.0(3.2)	83.5(3.7)	<0.001
*Row%*																
**Age group (years)**																
*50-54*	2644	36.0	60.7	3.3		17.9	82.1		65.0	18.5	16.5		36.0	53.0	11.1	
*55-59*	1892	52.7	44.5	2.7		39.1	60.9		69.8	17.2	12.9		39.0	44.2	16.8	
*60-64*	1133	64.9	32.7	2.5		51.5	48.5		73.8	14.7	11.6		46.5	34.6	18.9	
*65-69*	833	74.5	21.5	4.0		63.5	36.4		76.1	13.8	10.1		46.2	29.7	24.1	
*70-74*	621	82.8	13.4	3.9		75.7	24.3		75.0	13.7	11.3		44.8	28.3	26.9	
*75-79*	228	82.0	14.5	3.5		81.1	18.4		75.9	10.5	13.6		49.1	25.0	25.9	
*80+*	94	80.9	12.8	6.4	<0.001	81.9	18.1	<0.001	81.9	5.3	12.8	<0.001	37.2	34.0	28.7	<0.001
**Alcohol use**																
Never	3981	57.9	39.1	3.1		45.9	54.1		71.7	15.2	13.1		43.8	37.8	18.4	
<1/week	2811	49.8	47.1	3.2		33.8	66.2		67.4	18.1	14.5		36.7	48.4	14.9	
1-4/week	162	53.1	40.7	6.2		35.2	64.8		72.8	16.7	10.5		35.8	47.5	16.7	
5+/week	126	63.5	33.3	3.2		43.7	56.3		70.6	13.5	15.9		34.1	46.8	19.0	
Ex-drinker	196	62.8	34.7	2.6	<0.001	50.5	49.5	<0.001	78.1	11.2	10.7	0.001	44.4	35.2	20.4	<0.001
*Unknown*	169	54.4	40.8	4.7		41.4	57.4		68.6	17.8	13.6		36.1	43.2	20.7	
**Smoking status**																
*Never*	7192	54.5	42.4	3.1		40.1	59.9		70.0	16.4	13.5		40.4	42.5	17.1	
*Ex-smoker*	95	76.8	18.9	4.2		87.4	12.6		77.9	6.3	15.8		46.3	27.4	26.3	
*Current*	106	63.2	28.3	8.5	<0.001	65.1	34.9	<0.001	79.2	12.3	8.5	0.013	55.7	27.4	17.0	<0.001
*Unknown*	52	48.1	46.2	5.8		38.5	57.7		61.5	17.3	21.2		34.6	51.9	13.5	
**Physical activity**																
*Inactive*	626	55.4	40.7	3.8		42.8	56.9		71.4	15.8	12.8		40.3	38.0	21.7	
*Minimally active*	1848	54.9	42.0	3.1		41.5	58.5		70.1	17.4	12.6		41.7	41.3	16.9	
*HEPA active^$^*	4971	54.8	42.1	3.2	0.877	40.7	59.3	0.520	70.1	15.9	14.0	0.450	40.3	43.0	16.7	0.249

	**Socio-economic position in**															

		**Childhood(Parental possession of a watch, sewing machine, and bicycle**				Early Adulthood (Education*)			**Late Adulthood (Occupation)**				**Current (Household Income per Head)**			

	**No.**	**0 items**	**1+ items**	**Unknown**	***p *†**	**Low**	**High**	***p *†**	**Manual**	**Non-manual**	**Unknown**	***p *†**	**Low**	**High**	**Unknown**	***p *†**

***Men***																

Number of participants	2536	1664	812	60		730	1803		1581	791	164		1131	1035	370	
*Mean (SD)*																
White blood cells (10^9^/L)	2536	6.85(1.7)	6.86(1.7)	6.74(1.5)	0.878	7.01(1.7)	6.79(1.7)	0.010	6.87(1.7)	6.75(1.7)	7.15(1.9)	0.021	6.93(1.7)	6.73(1.7)	6.95(1.7)	0.012
Granulocytes (10^9^/L)	2536	4.21(1.4)	4.14(1.4)	4.17(1.1)	0.439	4.38(1.4)	4.11(1.3)	<0.001	4.21(1.4)	4.10(1.3)	4.40(1.6)	0.018	4.27(1.4)	4.07(1.3)	4.27(1.4)	0.002
Lymphocytes (10^9^/L)	2536	2.14(0.6)	2.23(0.7)	2.05(0.5)	0.002	2.10(0.6)	2.19(0.7)	0.006	2.16(0.7)	2.17(0.6)	2.23(0.6)	0.389	2.15(0.7)	2.18(0.6)	2.18(0.7)	0.619
Leg length (cm)	2519	75.2(3.8)	75.7(4.2)	75.3(3.7)	0.005	74.6(3.7)	75.6(4.0)	<0.001	75.2(3.9)	75.6(4.0)	75.6(3.9)	0.020	75.1(4.0)	75.7(3.8)	75.1(4.0)	0.003
Sitting height (cm)	2520	88.6(3.6)	89.8(3.4)	88.7(3.7)	<0.001	87.6(3.5)	89.6(3.4)	<0.001	88.8(3.6)	89.3(3.4)	89.7(4.0)	<0.001	88.5(3.5)	89.7(3.4)	88.6(3.8)	<0.001
*Row%*																
**Age group (years)**																
*50-54*	346	41.3	56.6	2.0		11.3	88.7		68.8	21.4	9.8		43.9	42.8	13.3	
*55-59*	568	57.0	41.0	1.9		23.6	76.4		67.3	25.4	7.4		44.9	43.0	12.1	
*60-64*	599	65.4	32.2	2.3		25.2	74.6		61.3	32.9	5.8		37.4	47.9	14.7	
*65-69*	470	76.0	22.3	1.7		30.6	69.1		56.8	38.5	4.7		48.5	37.0	14.5	
*70-74*	339	82.3	15.3	2.4		44.5	55.5		58.7	36.3	5.0		48.7	32.2	19.2	
*75-79*	156	78.2	16.0	5.8		51.3	48.1		60.9	32.1	7.1		48.1	35.9	16.0	
*80+*	58	81.0	13.8	5.2	<0.001	53.4	46.6	<0.001	56.9	37.9	5.2	<0.001	55.2	29.3	15.5	<0.001
**Alcohol use**																
Never	880	68.3	29.9	1.8		30.1	69.9		61.0	32.0	6.9		48.1	36.8	15.1	
<1/week	1068	63.2	34.7	2.1		24.4	75.6		61.5	32.0	6.5		41.5	44.4	14.1	
1-4/week	176	62.5	34.7	2.8		25.6	74.4		67.6	28.4	4.0		46.0	44.9	9.1	
5+/week	271	65.7	32.5	1.8		39.9	60.1		66.4	28.8	4.8		43.9	39.9	16.2	
Ex-drinker	93	71.0	21.5	7.5	0.046	37.6	62.4	<0.001	67.7	24.7	7.5	0.323	49.5	34.4	16.1	0.011
*Unknown*	48	70.8	18.8	10.4		33.3	60.4		52.1	33.3	14.6		39.6	37.5	22.9	
**Smoking status**																
*Never*	940	63.8	34.3	1.9		20.3	79.7		58.3	35.3	6.4		41.0	45.5	13.5	
*Ex-smoker*	675	69.0	28.1	2.8		33.5	66.5		62.2	31.3	6.5		46.2	40.4	13.3	
*Current*	900	65.1	32.9	2.0	0.039	34.3	65.7	<0.001	66.8	27.0	6.2	<0.001	47.6	36.2	16.2	<0.001
*Unknown*	21	57.1	19.0	23.8		19.0	66.7		57.1	23.8	19.0		28.6	38.1	33.3	
**Physical activity**																
*Inactive*	223	65.9	31.4	2.7		31.8	66.8		68.6	26.0	5.4		48.4	30.9	20.6	
*Minimally active*	866	61.1	36.4	2.5		29.1	70.9		58.8	33.9	7.3		44.3	40.9	14.8	
*HEPA active^$^*	1447	68.3	29.5	2.2	0.002	28.1	71.9	0.438	63.5	30.3	6.2	0.021	44.2	42.3	13.5	0.046

* Highest level of education was categorised as low (primary or less) and high (above primary)

# Manual is agricultural worker, factory worker or sales and service, non-manual is administrator/manager, professional/technical or military/disciplined

† p value for linear trend for continuous variables and *χ*^2 ^p value for categorical variables

*$ *Health enhancing physical activity: vigorous activity at least 3 days a week achieving at least 1500 metabolically equivalent tasks (MET) minutes per week or activity on 7 days of the week achieving at least 3000 MET minutes per week

Proxies of SEP were unavailable or unclassifiable for 28.7% of the participants, mainly because information on household income or the longest-held occupation was missing. Alcohol use or smoking was not available for 2% of participants. We used multiple imputation for missing data [28,29]. Socioeconomic position at any stage, alcohol use and smoking were predicted based on a flexible additive regression model with predictive mean matching incorporating age, sex, leg length, seated height, alcohol use, smoking status, physical activity and SEP at the other three stages [28]. We imputed missing values 10 times and analysed each complete dataset separately, then summarized estimates with confidence intervals adjusted for missing data uncertainty [30]. As a sensitivity analysis, a complete case analysis without imputation was performed. We used STATA version 10.0 (STATA Corp., College Station, TX) and R version 2.12.2 for analysis, imputation and model estimation.

## Results

Of the 10,088 phase 3 participants examined, 1.1% (*n *= 107) had missing data for total white blood cell, granulocyte or lymphocyte counts. Analysis was based on the remaining 9,981 participants. There were more women (*n *= 7,445) than men (*n *= 2,536) and the women were younger [mean age 59.2 years (S.D. 7.6)] than the men [mean age 63.1 years (S.D. 7.6)]. Overall the mean white blood cell and granulocyte counts were lower in women than men (Table 1).

The associations of SEP with white blood cell, granulocyte or lymphocyte counts did not vary with age (data not shown). However, associations of SEP with lymphocyte count varied with sex, so only sex stratified results have been presented for this outcome. For white blood cell count and granulocyte count, the sensitive periods model performed better than the fully saturated model, accumulation or critical period models (Table 2). The sensitive periods model shows that some life stages had stronger negative associations than others with white blood cell count and granulocyte count; the early adult stage had the strongest association for both outcomes.

**Table 2:** Associations of high socioeconomic position (compared to low) at four life stages with white cell count and its differentials, adjusted for age^a ^and sex in 9,981 Chinese adults (≥ 50 years) from the Guangzhou Biobank Cohort Study, phase 3, 2006-2008

		White Blood Cell Count (10^9^/L)			Granulocyte Cell Count (10^9^/L)	
	**Δ**	**95% CI**	**AIC**	**Δ**	**95% CI**	**AIC**

**Men and Women**						
Critical period Model^b^						
*Childhood*	**-0.14**	**(-0.20, -0.07)**	8995	**-0.10**	**(-0.15, -0.04)**	4095
*Early Adult*	**-0.35**	**(-0.42, -0.29)**	8909	**-0.26**	**(-0.31, -0.21)**	4018
*Late Adult*	**-0.21**	**(-0.29, -0.13)**	8980	**-0.17**	**(-0.23, -0.11)**	4074
*Current*	**-0.22**	**(-0.29, -0.15)**	8965	**-0.16**	**(-0.22, -0.11)**	4064
Accumulation Model*^c^*			8905			4012
*0*	0			0		
*1*	**-0.19**	**(-0.29, -0.09)**		**-0.14**	**(-0.22, -0.06)**	
*2*	**-0.32**	**(-0.42, -0.22)**		**-0.23**	**(-0.31, -0.16)**	
*3*	**-0.48**	**(-0.58, -0.38)**		**-0.35**	**(-0.43, -0.27)**	
*4*	**-0.51**	**(-0.65, -0.38)**		**-0.40**	**(-0.50, -0.29)**	
Sensitive Periods Model^d^			**8892**			**4000**
*Childhood*	-0.06	(-0.13, 0.01)		-0.03	(-0.09, 0.02)	
*Early Adult*	**-0.28**	**(-0.36, -0.20)**		**-0.20**	**(-0.26, -0.14)**	
*Late Adult*	-0.08	(-0.17, 0.002)		**-0.08**	**(-0.14, -0.01)**	
*Current*	**-0.12**	**(-0.19, -0.05)**		**-0.09**	**(-0.15, -0.04)**	
Fully Saturated Model^e^			8905			4015

Abbreviations: *CI *Confidence Interval; *AIC *Akaike Information Criterion

**Bold **numbers indicate P < 0.05 for coefficients or best fitting model for AICs

* Test for interaction with sex significant at p < 0.05 level

^a^Age in five year age groups. ^b^Estimates for SEP at each individual life stage adjusted for age and sex but unadjusted for SEP at other life stages. ^c^Number of periods in high SEP adjusted for age and sex. ^d^Mutually adjusted for SEP at other life stages and adjusted for age and sex. ^e^Includes SEP at all four life stages and all possible interactions between all four measures of SEP. Also adjusted for age and sex

The pattern for lymphocyte cell count was somewhat different. Associations varied by sex. Table 3 shows that for both sexes the accumulation and sensitive periods models did not perform as well as critical period models. The early adult life stage was a critical period for women, with a negative association between SEP and lymphocyte cell count. By contrast, for men, all estimates of association between SEP and lymphocyte cell count were positive, although all confidence intervals included zero.

**Table 3:** Associations of high socioeconomic position (compared to low) at four life stages with lymphocyte cell count (10^9^/L) by sex, adjusted for age^a ^in 9,981 Chinese adults (≥ 50 years) from the Guangzhou Biobank Cohort Study, phase 3, 2006-2008

	Women (n = 7,445)				Men (n = 2,536)	
	**Δ**	**95% CI**	**AIC**	**Δ**	**95% CI**	**AIC**

Critical period Model^b^						
*Childhood*	**-0.04**	**(-0.07**, **-0.01)**	-7874	0.03	(-0.03, 0.08)	-2277
*Early Adult*	**-0.08**	**(-0.11**, **-0.05)**	**-7894**	0.03	(-0.02, 0.09)	-2277
*Late Adult*	-0.03	(-0.07, 0.01)	-7871	0.04	(-0.01, 0.10)	**-2279**
*Current*	**-0.04**	**(-0.07**, **-0.01)**	-7874	0.01	(-0.05 0.07)	-2277
Accumulation Model*^c^*			-7885			-2274
*0*	0			0		
*1*	-0.02	(-0.07, 0.02)		0.02	(-0.07, 0.11)	
*2*	**-0.05**	**(-0.10**, **-0.01)**		0.05	(-0.03, 0.13)	
*3*	**-0.10**	**(-0.14**, **-0.05)**		0.04	(-0.05, 0.12)	
*4*	**-0.09**	**(-0.15**, **-0.03)**		0.10	(-0.01, 0.21)	
Sensitive Periods Model^d^			-7891			-2274
*Childhood*	-0.02	(-0.05, 0.01)		0.02	(-0.03, 0.08)	
*Early Adult*	**-0.07**	**(-0.10**, **-0.04)**		0.02	(-0.04, 0.08)	
*Late Adult*	0.00	(-0.04, 0.04)		0.04	(-0.02, 0.10)	
*Current*	-0.01	(-0.05, 0.02)		0.00	(-0.06, 0.06)	
Fully Saturated Model^e^			-7882			-2262

Abbreviations: *CI *Confidence Interval; *AIC *Akaike Information Criterion

**Bold **numbers indicate P < 0.05 for coefficients or best fitting model for AICs

^a^Age in five year age groups. ^b^Estimates for SEP at each individual life stage adjusted for age but unadjusted for SEP at other life stages. ^c^Number of periods in high SEP adjusted for age. ^d^Mutually adjusted for SEP at other life stages and adjusted for age ^e^Includes SEP at all four life stages and all possible interactions between all four measures of SEP. Also adjusted for age

Additional adjustment for lifestyle factors (smoking, alcohol use, and physical exercise) attenuated estimates slightly (see Appendix) but the pattern of associations generally remained the same. Smoking among men is associated with both low SEP and higher lymphocyte count, hence adjustment for smoking strengthened the positive association of SEP with lymphocyte count (Appendix). Further adjustment for BMI (Appendix) produced very similar results; estimates of association were little changed. All results were similar in a complete case analysis (Appendix).

## Discussion

Consistent with other studies in developed and developing settings examining the association between SEP and inflammation [5-11], we found that SEP was negatively associated with adult immune cell numbers, particularly among women. Consistent with the only other study from a developing country setting, the advantage of higher SEP for adult inflammation was less marked among men [10]. In general, considering SEP at all four life stages was better than considering individual life stages (critical periods) except for lymphocyte cell counts.

This study has a number of strengths. To our knowledge, it is the first study to investigate the role of life course SEP in later adulthood inflammation in a non-western, developing setting. Moreover, we explicitly determined the most parsimonious representation of life course SEP. The large sample size allowed sex-specific analysis. Nevertheless, there are limitations. First, it is a cross-sectional study with recalled SEP, which may be imprecise, although most likely non-differential. Second, in a cross-sectional design reverse causality must be considered although it is unlikely that inflammation has a causal effect on life course SEP. Third, there may have been gender bias in the allocation of resources within families, most likely favouring boys and men, which may have mitigated the disadvantages of low SEP. However it is unclear why this should have mitigated the effect of SEP for lymphocytes but not for white blood cells and granulocytes. Fourth, our cohort may not be fully population representative. However, prevalence of certain morbidities, such as diabetes, were similar to those in a representative sample of urban Chinese [31]. Fifth, survivor bias is possible, which may have limited participants' socioeconomic and health diversity, biasing results towards the null. If survivorship were an issue we would have expected differences in associations by age, of which there was no evidence. Sixth, we did not explicitly consider the life course effects of social mobility since these are particularly hard to define and test clearly. Inter- and intra-generational mobility, upward and downward mobility are all potential risk factors.

Seventh, a single measurement of white blood cells and differential cell counts may not accurately reflect long-term immune function or inflammation. However, white blood cell count is used as a marker of immune status in clinical settings and is a well-established and routinely-used marker of systemic inflammation [32]. White blood cell count is associated with disease risk and predicts disease outcome [33,34]. Eighth, although we report associations between SEP and differential white blood cell counts, clinical significance remains to be determined. Within the normal range, elevated white blood cell counts are associated with risk factors for chronic diseases, such as cardiovascular disease [32,35]. White blood cell counts can be conceptualised as a mixed marker of exposure and response, even a relatively small shift towards a healthier inflammation-immunological profile might have significant public health benefits at the population level [33,34]. Ninth, acute infection, trauma and underlying chronic disease or medication could be mediators. There is no evidence to suggest that participants were experiencing infection during the assessment process, nor significant trauma. Although only those with life-threatening illness were specifically excluded, those experiencing significant acute infection or trauma were less likely to attend this study, which should have minimized any bias from this source. We also performed descriptive analysis of the data to detect and exclude outliers, which may have resulted from unknown underlying disease, medication, or recording error.

One possible explanation for the association of low SEP with inflammation is via current health behaviour linked to inflammation [5,36-38]. Although we did not perform formal tests of mediation, we did adjust for smoking, alcohol consumption, physical activity and BMI in separate models (Appendix), which had little effect among women, but among men, this attenuated the negative association of early adult SEP with white blood cell and granulocyte counts and strengthened the positive association of early adult SEP with lymphocyte counts. This suggests that any associations are unlikely to be driven by adult health behaviour in women, though these may obscure negative associations of early adult SEP with inflammatory markers in men.

Low SEP may increase exposure to pro-inflammatory agents, such as microbial pathogens, pollutants or adverse work conditions. Mechanisms for increased exposure or vulnerability to pathogens in low SEP groups include earlier and/or greater lifetime exposure due to adverse living conditions, such as overcrowding, and increased susceptibility to primary infection through nutritional deficiencies, or stress-related immune dysfunction [3]. A gender bias may have protected low SEP men from such exposures and adverse work conditions, although it is not clear why the effects should be most obvious for lymphocytes. Lower birth weight amongst those with low childhood SEP is another possible explanation, but birth weight is not available for our participants. Birth weight is inversely associated with inflammatory markers [6,39]. However, birth weight appears to be less relevant in developing country settings such as ours [40], and there is no reason why birth weight should have sex-specific effects on some white cell sub-types.

An alternative explanation is that better early life conditions would be expected to promote development of the adaptive immune system, particularly of the thymus,[41] whose development takes place in early life [41] and which is sensitive to malnutrition, micro-nutrient deficiencies and infections during growth and development [42-44]. Moreover, the same exposure would also allow upregulation of the gonadotropic axis resulting in sex-specific effects on some immune cell sub-populations [45-47], particularly those relating to adaptive immunity. Consistent with this mechanism we have previously observed similar sex-specific associations, in the Guangzhou Biobank Cohort Study, of childhood stress with white cell count [48] and of childhood diet with lymphocytes but not granulocytes [19]. However, we do not have measurements that would allow proof of this mechanism.

## Conclusions

Socioeconomic position was inversely associated with white blood cell differential counts, as a marker of inflammation, with a clearer and more consistent association among women than men. Environmentally and inter-generationally driven changes to the gonadotropic axis may obscure the normally protective effect of social advantage in the first few generations of men, but not women, to experience better living conditions. Given the links between the immune system, inflammation and chronic disease, this provides a biological mechanism between SEP and the pathophysiological genesis of chronic disease. Understanding such mechanisms for populations experiencing the epidemiological transition is of public health significance.

## Competing interests

The authors declare that they have no competing interests.

## Authors' contributions

DA West carried out the statistical analyses and wrote the article. TM Elwell-Sutton performed additional analysis and reviewed drafts of the manuscript. CM Schooling and GM Leung helped conceptualize ideas, interpret findings and review drafts of the manuscript. TH Lam, CQ Jiang and KK Cheng initiated and oversee the Guangzhou Biobank Cohort Study and WS Zhang assisted in the planning and co-ordination of the study. All authors read and approved the final manuscript.

## Appendix

Associations of socioeconomic position with markers of inflammation, additionally adjusted for lifestyles factors (smoking, alcohol use and physical exercise), are shown below: associations with white cell counts and granulocyte counts are shown in Table 4 and sex-specific associations with lymphocyte counts are shown in Table 5. Associations further adjusted for body mass index are shown in Table 6 (for white cell count and granulocyte count) and Table 7 (lymphocyte count). Results from the complete case analysis are shown in below in Table 8 (white cell count and granulocyte count) and Table 9 (lymphocyte count).

**Table 4:** Associations of high socioeconomic position compared to low at four life stages with white cell count and granulocyte count, adjusted for age^a^, sex and lifestyle factors in 9,981 Chinese adults (≥50 years) from the Guangzhou Biobank Cohort Study, phase 3, 2006-2008

	White Blood Cell Count (10^9^/L)			Granulocyte Cell Count (10^9^/L)		
	**Δ**	**95% CI**	**AIC**	**Δ**	**95% CI**	**AIC**

**Men and Women**						
Critical Period Model^b^						
*Childhood*	**-0.13**	**(-0.19**, **-0.06)**	8789	**-0.09**	**(-0.14**, **-0.04)**	3967
*Early Adult*	-0.31*	**(-0.38**, **-0.24)**	8725	**-0.23**	**(-0.29**, **-0.18)**	3907
*Late Adult*	-0.18*	**(-0.27**, **-0.10)**	8779	**-0.16**	**(-0.22**, **-0.09)**	3951
*Current*	**-0.18**	**(-0.25**, **-0.12)**	8769	**-0.15**	**(-0.20**, **-0.09)**	3944
Accumulation Model*^c^*			8722			3901
*0*	0			0		
*1*	**-0.17**	**(-0.27**, **-0.07)**		**-0.13**	**(-0.21**, **-0.05)**	
*2*	**-0.28**	**(-0.37**, **-0.18)**		**-0.21**	**(-0.28**, **-0.13)**	
*3*	**-0.42**	**(-0.52**, **-0.32)**		**-0.32**	**(-0.40**, **-0.24)**	
*4*	**-0.46**	**(-0.59**, **-0.32)**		**-0.37**	**(-0.47**, **-0.26)**	
Sensitive Periods Model^d^			**8713**			**3892**
*Childhood*	-0.06	(-0.13, 0.01)		-0.04	(-0.09, 0.02)	
*Early Adult*	**-0.24**	**(-0.32**, **-0.17)**		**-0.18**	**(-0.24**, **-0.12)**	
*Late Adult*	-0.08	(-0.16, 0.01)		**-0.07**	**(-0.14**, **-0.01)**	
*Current*	**-0.10**	**(-0.18**, **-0.03)**		**-0.08**	**(-0.14**, **-0.02)**	
Fully Saturated Model^e^			8725			3907

Abbreviations: *CI *Confidence Interval; *AIC *Akaike Information Criterion

**Bold **numbers indicate P < 0.05 for coefficients or best fitting model for AICs * Test for interaction with sex significant at p < 0.05 level

^a^Age in five year age groups. ^b^Estimates for SEP at each individual life stage adjusted for age, sex, smoking, alcohol use, and physical activity but unadjusted for SEP at other life stages. ^c^Number of periods in high SEP adjusted for age, sex, smoking, alcohol use, and physical activity. ^d^Mutually adjusted for SEP at other life stages and adjusted for age, sex, smoking, alcohol use, and physical activity. ^e^Includes SEP at all four life stages and all possible interactions between all four measures of SEP. Also adjusted for age, sex, smoking, alcohol use, and physical activity

**Table 5:** Associations of high socioeconomic position (compared to low) at four life stages with lymphocyte cell count (10^9^/L) by sex, adjusted for age^a ^and lifestyle factors in 9,981 Chinese adults (≥50 years) from the Guangzhou Biobank Cohort Study, phase 3, 2006-2008

	Women (n = 7,445)				Men (n = 2,536)	
	**Δ**	**95% CI**	**AIC**	**Δ**	**95% CI**	**AIC**

Critical period Model^b^						
*Childhood*	**-0.03**	**(-0.06, -0.01)**	-7876	0.03	(-0.02, 0.09)	-2354
*Early Adult*	**-0.08**	**(-0.11, -0.05)**	**-7894**	**0.07**	**(0.02, 0.13)**	**-2360**
*Late Adult*	-0.03	(-0.07, 0.01)	-7873	**0.06**	**(0.003, 0.11)**	-2358
*Current*	**-0.03**	**(-0.06, -0.001)**	-7876	0.03	(-0.02, 0.09)	-2355
Accumulation Model*^c^*			-7885			-2357
*0*	0			0		
*1*	-0.02	(-0.07, 0.02)		0.04	(-0.05, 0.12)	
*2*	**-0.05**	**(-0.10, -0.01)**		**0.08**	**(0.004, 0.16)**	
*3*	**-0.09**	**(-0.13, -0.04)**		**0.08**	**(0.001, 0.17)**	
*4*	**-0.09**	**(-0.14, -0.03)**		**0.15**	**(0.03, 0.26)**	
Sensitive Periods Model^d^			-7891			-2357
*Childhood*	-0.02	(-0.05, 0.01)		0.02	(-0.03, 0.08)	
*Early Adult*	**-0.07**	**(-0.10, -0.03)**		0.06	(-0.01, 0.12)	
*Late Adult*	0.00	(-0.04, 0.04)		0.04	(-0.02, 0.10)	
*Current*	-0.01	(-0.04, 0.02)		0.01	(-0.05, 0.07)	
Fully Saturated Model^e^			-7881			-2345

Abbreviations: *CI *Confidence Interval; *AIC *Akaike Information Criterion

**Bold **numbers indicate P < 0.05 for coefficients or best fitting model for AICs

^a^Age in five year age groups. ^b^Estimates for SEP at each individual life stage adjusted for age, smoking, alcohol use, and physical activity but unadjusted for SEP at other life stages. ^c^Number of periods in high SEP adjusted for age, smoking, alcohol use, and physical activity. ^d^Mutually adjusted for SEP at other life stages and adjusted for age, smoking, alcohol use, and physical activity. ^e^Includes SEP at all four life stages and all possible interactions between all four measures of SEP. Also adjusted for age, smoking, alcohol use, and physical activity

**Table 6:** Associations of high socioeconomic position (compared to low) at four life stages with white cell count and granulocyte count, adjusted for age^a^, sex and lifestyle factors and body mass index in 9,981 Chinese adults (≥50 years) from the Guangzhou Biobank Cohort Study, phase 3, 2006-2008

		White Blood Cell Count (10^9^/L)			Granulocyte Cell Count (10^9^/L)	
	**Δ**	**95% CI**	**AIC**	**Δ**	**95% CI**	**AIC**

**Men and Women**						
Critical period Model^b^						
*Childhood*	**-0.13**	**(-0.19**, **-0.06)**	8248	**-0.09**	**(-0.14**, **-0.04)**	3675
*Early Adult*	**-0.31**	**(-0.38**, **-0.24)**	8206	**-0.24**	**(-0.29**, **-0.18)**	3631
*Late Adult*	**-0.20**	**(-0.27**, **-0.12)**	8239	**-0.17**	**(-0.23**, **-0.10)**	3659
*Current*	**-0.20**	**(-0.27**, **-0.14)**	8223	**-0.16**	**(-0.21**, **-0.11)**	3646
Accumulation Model*^c^*						3611
*0*	0		8186.0	0		
*1*	**-0.19**	**(-0.29**, **-0.09)**		**-0.14**	**(-0.22**, **-0.06)**	
*2*	**-0.28**	**(-0.37**, **-0.18)**		**-0.21**	**(-0.29**, **-0.14)**	
*3*	**-0.40**	**(-0.50**, **-0.30)**		**-0.31**	**(-0.39**, **-0.23)**	
*4*	**-0.42**	**(-0.56**, **-0.29)**		**-0.35**	**(-0.45**, **-0.24)**	
Sensitive Periods Model^d^			**8185.9**			**3608**
*Childhood *	-0.06	(-0.13, 0.01)		-0.04	(-0.09, 0.02)	
*Early Adult *	**-0.18**	**(-0.25**, **-0.10)**		**-0.14**	**(-0.20**, **-0.08)**	
*Late Adult *	-0.08	(-0.16, 0.00)		**-0.08**	**(-0.14**, **-0.02)**	
*Current *	**-0.12**	**(-0.20**, **-0.05)**		**-0.10**	**(-0.15**, **-0.05)**	
Fully Saturated Model^e^			8198			3624

Abbreviations: *CI *Confidence Interval; *AIC *Akaike Information Criterion

**Bold **numbers indicate P < 0.05 for coefficients or best fitting model for AICs

^a^Age in five year age groups. ^b^Estimates for SEP at each individual life stage adjusted for age, sex, smoking, alcohol use, and physical activity and body mass index but unadjusted for SEP at other life stages. ^c^Number of periods in high SEP adjusted for age, sex, smoking, alcohol use, and physical activity and body mass index. ^d^Mutually adjusted for SEP at other life stages and adjusted for age, sex, smoking, alcohol use, and physical activity and body mass index. ^e^Includes SEP at all four life stages and all possible interactions between all four measures of SEP. Also adjusted for age, sex, smoking, alcohol use, and physical activity

**Table 7:** Associations of high socioeconomic position (compared to low) at four life stages with lymphocyte cell count (10^9^/L) by sex, adjusted for age^a^, lifestyle factors and body mass index in 9,981 Chinese adults(≥50 years) from the Guangzhou Biobank Cohort Study, phase3, 2006-2008

	Women (n = 7,445)				Men (n = 2,536)	
	**Δ**	**95% CI**	**AIC**	**Δ**	**95% CI**	**AIC**

Critical period Model^b^						
*Childhood *	**-0.03**	**(-0.06**, **-0.01)**	-8283	0.03	(-0.02, 0.09)	-2438
*Early Adult *	**-0.08**	**(-0.11**, **-0.05)**	**-8288**	**0.07**	**(0.02**, **0.13)**	**-2443**
*Late Adult *	-0.03	(-0.06, 0.01)	-8278	**0.05**	**(0.00**, **0.11)**	-2440
*Current *	**-0.03**	**(-0.06**, **-0.01)**	-8281	0.03	(-0.02, 0.09)	-2438
Accumulation Model*^c^*			-8284			-2439
*0*	0*			0*		
*1 *	-0.03	(-0.08, 0.01)		0.02	(-0.06, 0.10)	
*2 *	-0.04	(-0.08, 0.002)		0.07	(-0.01, 0.15)	
*3 *	**-0.07**	**(-0.12**, **-0.02)**		0.07	(-0.02, 0.15)	
*4 *	**-0.06**	**(-0.12**, **-0.0003)**		**0.13**	**(0.02**, **0.24)**	
Sensitive Periods Model^d^			-8285			-2440
*Childhood *	-0.02	(-0.05, 0.01)		0.02	(-0.04, 0.08)	
*Early Adult *	**-0.04**	**(-0.07**, **-0.01)**		**0.06**	**(0.002**, **0.12)**	
*Late Adult *	0.01	(-0.03, 0.05)		0.03	(-0.03, 0.09)	
*Current *	-0.01	(-0.04, 0.02)		0.00	(-0.06, 0.06)	
Fully Saturated Model^e^			-8274			-2428

Abbreviations: *CI *Confidence Interval; *AIC *Akaike Information Criterion

**Bold **numbers indicate P < 0.05 for coefficients or best fitting model for AICs

^a^Age in five year age groups. ^b^Estimates for SEP at each individual life stage adjusted for age, smoking, alcohol use, and physical activity and body mass index but unadjusted for SEP at other life stages. ^c^Number of periods in high SEP adjusted for age, smoking, alcohol use, and physical activity and body mass index. ^d^Mutually adjusted for SEP at other life stages and adjusted for age, smoking, alcohol use, and physical activity and body mass index. ^e^Includes SEP at all four life stages and all possible interactions between all four measures of SEP. Also adjusted for age, smoking, alcohol use, and physical activity

**Table 8:** Associations of high socioeconomic position (compared to low) at four life stages with white cell count and its differentials, adjusted for age^a ^and sex Chinese adults (≥50 years) from the Guangzhou Biobank Cohort Study, phase 3, 2006-2008 including complete cases only

		White Blood Cell Count (10^9^/L)				Granulocyte Cell Count (10^9^/L)		
	**N**	**Δ**	**95% CI**	**AIC**	**N**	**Δ**	**95% CI**	**AIC**

**Men and Women**								
Critical period Model^b^								
*Childhood *	9683	**-0.14**	**(-0.21**, **-0.07)**	8769	9683	**-0.10**	**(-0.15**, **-0.04)**	4038
*Early Adult *	9976	**-0.35**	**(-0.42**, **-0.29)**	8906	9976	**-0.26**	**(-0.31**, **-0.21)**	4018
*Late Adult *	8808	**-0.23**	**(-0.31**, **-0.15)**	7901	8808	**-0.19**	**(-0.25**, **-0.12)**	3575
*Current *	8332	**-0.23**	**(-0.30**, **-0.16)**	7446	8332	**-0.17**	**(-0.23**, **-0.12)**	3362
Accumulation Model*^c^*				6327				2872
*0*	7117	0			7117	0.00		
*1 *	7117	**-0.19**	**(-0.31**, **-0.07)**		7117	**-0.18**	**(-0.28**, **-0.08)**	
*2 *	7117	**-0.38**	**(-0.50**, **-0.26)**		7117	**-0.30**	**(-0.40**, **-0.21)**	
*3 *	7117	**-0.54**	**(-0.66**, **-0.42)**		7117	**-0.43**	**(-0.52**, **-0.33)**	
*4 *	7117	**-0.53**	**(-0.69**, **-0.38)**		7117	**-0.42**	**(-0.55**, **-0.30)**	
Sensitive Periods Model^d^				6299				2851
*Childhood *	7112	-0.05	(-0.13, 0.03)		7112	-0.03	(-0.09, 0.04)	
*Early Adult *	7112	**-0.35**	**(-0.44**, **-0.26)**		7112	**-0.25**	**(-0.32**, **-0.18)**	
*Late Adult *	7112	-0.07	(-0.16, 0.02)		7112	-0.07	(-0.14, -0.002)	
*Current *	7112	**-0.12**	**(-0.20**, **-0.05)**		7112	**-0.10**	**(-0.16**, **-0.03)**	
Fully Saturated Model ^e^	7112			6310	7112			2864

Abbreviations: *CI *Confidence Interval; *AIC *Akaike Information Criterion

**Bold **numbers indicate P < 0.05 for coefficients or best fitting model for AICs

* Test for interaction with sex significant at p < 0.05 level

^a^Age in five year age groups. ^b^Estimates for SEP at each individual life stage adjusted for age and sex but unadjusted for SEP at other life stages. ^c^Number of periods in high SEP adjusted for age and sex. ^d^Mutually adjusted for SEP at other life stages and adjusted for age and sex. ^e^Includes SEP at all four life stages and all possible interactions between all four measures of SEP. Also adjusted for age and sex

**Table 9:** Associations of high socioeconomic position (compared to low) at four life stages with lymphocyte cell count (10^9^/L) by sex, adjusted for age^a ^in 9,981 Chinese adults (≥50 years) from the Guangzhou Biobank Cohort Study, phase 3, 2006-2008 including complete cases only

			Women (n = 7,445)				Men (n = 2,536)	
	**N**	**Δ**	**95% CI**	**AIC**	**N**	**Δ**	**95% CI**	**AIC**

Critical period Model^b^								
*Childhood*	7207	**-0.04**	**(-0.07**, **-0.01)**	-7619	2476	0.03	(-0.03, 0.08)	-2206
*Early Adult*	7443	**-0.08**	**(-0.11**, **-0.05)**	-7893	2533	0.03	(-0.02, 0.09)	-2274
*Late Adult*	6436	-0.03	(-0.07, 0.01)	-6835	2372	0.04	(-0.02, 0.09)	-2118
*Current*	6166	**-0.04**	**(-0.07**, **-0.01)**	-6589	2166	0.01	(-0.05, 0.06)	-1982
Accumulation Model*^c^*				-5520				-1783
0		0.00			1981	0.00		
*1*	5136	0.02	(-0.04, 0.07)		1981	0.03	(-0.08, 0.13)	
*2*	5136	-0.04	(-0.10, 0.01)		1981	0.07	(-0.03, 0.16)	
*3*	5136	**-0.10**	**(-0.15**, **-0.04)**		1981	0.08	(-0.02, 0.17)	
*4*	5136	**-0.07**	**(-0.14**, **-0.002)**		1981	0.07	(-0.05, 0.20)	
Sensitive Periods Model^d^				-5512				-1782
*Childhood*	5131	-0.03	(-0.06, 0.01)		1981	0.03	(-0.03, 0.09)	
*Early Adult*	5131	**-0.08**	**(-0.12**, **-0.04)**		1981	-0.01	(-0.08, 0.06)	
*Late Adult*	5131	0.02	(-0.03, 0.06)		1981	0.03	(-0.03, 0.09)	
*Current*	5131	-0.01	(-0.05, 0.02)		1981	-0.01	(-0.06, 0.05)	
Fully Saturated Model^e^	5131			-7222	1981			-7222

Abbreviations: *CI *Confidence Interval; *AIC *Akaike Information Criterion

**Bold **numbers indicate P < 0.05 for coefficients or best fitting model for AICs

^a^Age in five year age groups. ^b^Estimates for SEP at each individual life stage adjusted for age but unadjusted for SEP at other life stages. ^c^Number of periods in high SEP adjusted for age. ^d^Mutually adjusted for SEP at other life stages and adjusted for age. ^e^Includes SEP at all four life stages and all possible interactions between all four measures of SEP. Also adjusted for age

## Pre-publication history

The pre-publication history for this paper can be accessed here:

http://www.biomedcentral.com/1471-2458/12/269/prepub

## References

[B1] Pollitt RA Rose KM Kaufman JS Evaluating the evidence for models of life course socioeconomic factors and cardiovascular outcomes: a systematic review BMC Publ Health 2005 5 7 10.1186/1471-2458-5-7 PMC548689 15661071

[B2] Galobardes B Smith GD Lynch JW Systematic review of the influence of childhood socioeconomic circumstances on risk for cardiovascular disease in adulthood Ann Epidemiol 2006 16 2 91 104 10.1016/j.annepidem.2005.06.053 16257232

[B3] Simanek AM Dowd JB Aiello AE Persistent pathogens linking socioeconomic position and cardiovascular disease in the US Int J Epidemiol 2009 38 3 775 787 10.1093/ije/dyn273 19109247 PMC2689394

[B4] Steptoe A Shamaei-Tousi A Gylfe A Henderson B Bergstrom S Marmot M Socioeconomic status, pathogen burden and cardiovascular disease risk Heart 2007 93 12 1567 1570 17488763 10.1136/hrt.2006.113993 PMC2095775

[B5] Pollitt RA Kaufman JS Rose KM Diez-Roux AV Zeng D Heiss G Early-life and adult socioeconomic status and inflammatory risk markers in adulthood Eur J Epidemiol 2007 22 1 55 66 10.1007/s10654-006-9082-1 17225957

[B6] Jousilahti P Salomaa V Rasi V Vahtera E Palosuo T Association of markers of systemic inflammation, C reactive protein, serum amyloid A, and fibrinogen, with socioeconomic status J Epidemiol Community Health 2003 57 9 730 733 10.1136/jech.57.9.730 12933781 PMC1732590

[B7] Tabassum F Kumari M Rumley A Lowe G Power C Strachan DP Effects of socioeconomic position on inflammatory and hemostatic markers: a life-course analysis in the 1958 British birth cohort Am J Epidemiol 2008 167 11 1332 1341 10.1093/aje/kwn055 18367468

[B8] Gimeno D Ferrie JE Elovainio M Pulkki-Raback L Keltikangas-Jarvinen L Eklund C Hurme M Lehtimaki T Marniemi J Viikari JS When do social inequalities in C-reactive protein start? A life course perspective from conception to adulthood in the Cardiovascular Risk in Young Finns Study Int J Epidemiol 2008 37 2 290 298 10.1093/ije/dym244 18056120

[B9] Loucks EB Pilote L Lynch JW Richard H Almeida ND Benjamin EJ Murabito JM Life course socioeconomic position is associated with inflammatory markers: the Framingham Offspring Study Soc Sci Med 2010 71 1 187 195 10.1016/j.socscimed.2010.03.012 20430502 PMC2895737

[B10] Nazmi A Oliveira IO Horta BL Gigante DP Victora CG Lifecourse socioeconomic trajectories and C-reactive protein levels in young adults: findings from a Brazilian birth cohort Soc Sci Med 2010 70 8 1229 1236 10.1016/j.socscimed.2009.12.014 20137842 PMC2877874

[B11] Pollitt RA Kaufman JS Rose KM Diez-Roux AV Zeng D Heiss G Cumulative life course and adult socioeconomic status and markers of inflammation in adulthood J Epidemiol Community Health 2008 62 6 484 491 10.1136/jech.2006.054106 18477746

[B12] McDade TW Beck MA Kuzawa C Adair LS Prenatal undernutrition, postnatal environments, and antibody response to vaccination in adolescence Am J Clin Nutr 2001 74 4 543 548 11566655 10.1093/ajcn/74.4.543

[B13] McDade TW Life history, maintenance, and the early origins of immune function Am J Hum Biol: the official Journal of the Human Biology Council 2005 17 1 81 94 10.1002/ajhb.20095 15612049

[B14] Kuh D Ben-Shlomo Y A life course approach to chronic disease epidemiology 2004 2 New York: Oxford University Press 11980781

[B15] McDade TW The ecologies of human immune function Annu Rev Anthropol 2005 34 1 495 521 10.1146/annurev.anthro.34.081804.120348

[B16] Bogin B Silva MI Rios L Life history trade-offs in human growth: adaptation or pathology? Am J Hum Biol 2007 19 5 631 642 10.1002/ajhb.20666 17636530

[B17] Schooling CM Jiang CQ Lam TH Zhang WS Cheng KK Leung GM Life-course origins of social inequalities in metabolic risk in the population of a developing country Am J Epidemiol 2008 167 4 419 428 10.1093/aje/kwm329 18056924

[B18] Schooling CM Leung GM A socio-biological explanation for social disparities in non-communicable chronic diseases: the product of history? J Epidemiol Community Health 2010 64 11 941 949 10.1136/jech.2008.086553 20515893

[B19] Schooling CM Jiang CQ Lam TH Zhang WS Cheng KK Leung GM Childhood meat eating and inflammatory markers: the Guangzhou Biobank Cohort Study BMC Publ Health 2011 11 345 10.1186/1471-2458-11-345 PMC3121633 21595911

[B20] Jiang C Thomas GN Lam TH Schooling CM Zhang W Lao X Adab P Liu B Leung GM Cheng KK Cohort profile: the Guangzhou Biobank Cohort Study, a Guangzhou-Hong Kong-Birmingham collaboration Int J Epidemiol 2006 35 4 844 852 10.1093/ije/dyl131 16844769

[B21] Kavikondala S Jiang CQ Zhang WS Cheng KK Lam TH Leung GM Schooling CM Intergenerational [']mismatch' and adiposity in a developing population: the Guangzhou biobank cohort study Soc Sci Med 2010 70 6 834 843 10.1016/j.socscimed.2009.11.009 20079564

[B22] Langenberg C Kuh D Wadsworth ME Brunner E Hardy R Social circumstances and education: life course origins of social inequalities in metabolic risk in a prospective national birth cohort Am J Public Health 2006 96 12 2216 2221 10.2105/AJPH.2004.049429 17077402 PMC1698170

[B23] Taylor B Tofler G Morel-Kopp MC Carey H Carter T Elliott M Dailey C Villata L Ward C Woodward M The effect of initial treatment of periodontitis on systemic markers of inflammation and cardiovascular risk: a randomized controlled trial Eur J Oral Sci 2010 118 4 350 356 10.1111/j.1600-0722.2010.00748.x 20662907

[B24] Dawczynski C Schubert R Hein G Muller A Eidner T Vogelsang H Basu S Jahreis G Long-term moderate intervention with n-3 long-chain PUFA-supplemented dairy products: effects on pathophysiological biomarkers in patients with rheumatoid arthritis Br J Nutr 2009 101 10 1517 1526 10.1017/S0007114508076216 19245735

[B25] Mishra G Nitsch D Black S De Stavola B Kuh D Hardy R A structured approach to modelling the effects of binary exposure variables over the life course Int J Epidemiol 2009 38 2 528 537 19028777 10.1093/ije/dyn229 PMC2663717

[B26] Elwell-Sutton TM Jiang CQ Zhang WS Cheng KK Lam TH Leung GM Schooling CM Socioeconomic influences at different life stages on health in Guangzhou, China Soc Sci Med 2011 72 11 1884 1892 10.1016/j.socscimed.2011.03.041 21550152

[B27] Akaike H A new look at the statistical model identification IEEE Trans Autom Control 1974 19 6 716 723 10.1109/TAC.1974.1100705

[B28] Harrell FE Regression modeling strategies: with applications to linear models, logistic regression, and survival analysis 2001 New York: Springer

[B29] Schafer JL Multiple imputation: a primer Stat Methods Med Res 1999 8 1 3 15 10.1191/096228099671525676 10347857

[B30] Schafer JL Olsen MK Multiple imputation for multivariate missing-data problems: a data analyst's perspective Multivar Behav Res 1998 33 4 545 571 10.1207/s15327906mbr3304_5 26753828

[B31] Gu D Reynolds K Duan X Xin X Chen J Wu X Mo J Whelton PK He J Prevalence of diabetes and impaired fasting glucose in the Chinese adult population: International Collaborative Study of Cardiovascular Disease in Asia (InterASIA) Diabetologia 2003 46 9 1190 1198 10.1007/s00125-003-1167-8 12879248

[B32] Hoffman M Blum A Baruch R Kaplan E Benjamin M Leukocytes and coronary heart disease Atherosclerosis 2004 172 1 1 6 10.1016/S0021-9150(03)00164-3 14709350

[B33] Jee SH Park JY Kim HS Lee TY Samet JM White blood cell count and risk for all-cause, cardiovascular, and cancer mortality in a cohort of Koreans Am J Epidemiol 2005 162 11 1062 1069 10.1093/aje/kwi326 16221804

[B34] Kannel WB Anderson K Wilson PW White blood cell count and cardiovascular disease. Insights from the Framingham Study JAMA: the J Am Med Assoc 1992 267 9 1253 1256 10.1001/jama.1992.03480090101035 1538564

[B35] Madjid M Awan I Willerson JT Casscells SW Leukocyte count and coronary heart disease: implications for risk assessment J Am Coll Cardiol 2004 44 10 1945 1956 10.1016/j.jacc.2004.07.056 15542275

[B36] Lynch JW Kaplan GA Salonen JT Why do poor people behave poorly? Variation in adult health behaviours and psychosocial characteristics by stages of the socioeconomic lifecourse Soc Sci Med 1997 44 6 809 819 10.1016/S0277-9536(96)00191-8 9080564

[B37] Frohlich M Sund M Lowel H Imhof A Hoffmeister A Koenig W Independent association of various smoking characteristics with markers of systemic inflammation in men. Results from a representative sample of the general population (MONICA Augsburg Survey 1994/95) European Heart Journal 2003 24 14 1365 1372 10.1016/S0195-668X(03)00260-4 12871694

[B38] Koster A Bosma H Penninx BW Newman AB Harris TB van Eijk JT Kempen GI Simonsick EM Johnson KC Rooks RN Association of inflammatory markers with socioeconomic status The J Gerontol Series A, Biologic Sci Medi Sci 2006 61 3 284 290 10.1093/gerona/61.3.284 16567379

[B39] Kramer MS Seguin L Lydon J Goulet L Socio-economic disparities in pregnancy outcome: why do the poor fare so poorly? Paediatr Perinat Epidemiol 2000 14 3 194 210 10.1046/j.1365-3016.2000.00266.x 10949211

[B40] Victora CG Adair L Fall C Hallal PC Martorell R Richter L Sachdev HS Maternal and child undernutrition: consequences for adult health and human capital Lancet 2008 371 9609 340 357 10.1016/S0140-6736(07)61692-4 18206223 PMC2258311

[B41] Lynch HE Goldberg GL Chidgey A Van den Brink MR Boyd R Sempowski GD Thymic involution and immune reconstitution Trends Immunol 2009 30 7 366 373 10.1016/j.it.2009.04.003 19540807 PMC2750859

[B42] Hollander GA Krenger W Blazar BR Emerging strategies to boost thymic function Curr Opin Pharmacol 2010 10 4 443 453 10.1016/j.coph.2010.04.008 20447867 PMC3123661

[B43] Savino W Dardenne M Velloso LA Dayse-Silva-Barbosa S The thymus is a common target in malnutrition and infection The Brit J Nutri 2007 98 Suppl 1 S11 16 10.1017/S0007114507832880 17922946

[B44] McDade TW Beck MA Kuzawa CW Adair LS Prenatal undernutrition and postnatal growth are associated with adolescent thymic function J Nutr 2001 131 4 1225 1231 11285331 10.1093/jn/131.4.1225

[B45] Tanriverdi F Silveira LF MacColl GS Bouloux PM The hypothalamic-pituitary-gonadal axis: immune function and autoimmunity J Endocrinol 2003 176 3 293 304 10.1677/joe.0.1760293 12630914

[B46] Stygar D Westlund P Eriksson H Sahlin L Identification of wild type and variants of oestrogen receptors in polymorphonuclear and mononuclear leucocytes Clin Endocrinol 2006 64 1 74 81 10.1111/j.1365-2265.2005.02420.x 16402932

[B47] McMurray RW Suwannaroj S Ndebele K Jenkins JK Differential effects of sex steroids on T and B cells: modulation of cell cycle phase distribution, apoptosis and bcl-2 protein levels Pathobiology 2001 69 1 44 58 10.1159/000048757 11641617

[B48] Schooling CM Jiang C Lam TH Zhang W Cheng KK Leung GM Parental death during childhood and adult cardiovascular risk in a developing country: the Guangzhou Biobank Cohort Study PLoS One 2011 6 5 e19675 10.1371/journal.pone.0019675 21603607 PMC3095611

